# Thematic Analysis of Military Medical Ethics Publications From 2000 to 2020—A Bibliometric Approach

**DOI:** 10.1093/milmed/usab317

**Published:** 2021-07-31

**Authors:** Zachary Bailey, Peter Mahoney, Marina Miron, Martin Bricknell

**Affiliations:** U.S. Military Graduate Education, Air Force Institute of Technology—Civilian Institute, Wright-Patterson Air Force Base, OH 45433-7765, USA; Conflict and Health Research Group, King’s College London, Strand, London WC2R 2LS, UK; Centre for Military Ethics, Defence Academy, Swindon SN6 8LA, UK; Conflict and Health Research Group, King’s College London, Strand, London WC2R 2LS, UK; Centre for Military Ethics, Defence Academy, Swindon SN6 8LA, UK; Conflict and Health Research Group, King’s College London, Strand, London WC2R 2LS, UK; Centre for Military Ethics, Defence Academy, Swindon SN6 8LA, UK; Conflict and Health Research Group, King’s College London, Strand, London WC2R 2LS, UK; Centre for Military Ethics, Defence Academy, Swindon SN6 8LA, UK

## Abstract

**Introduction:**

There has been external criticism of the compliance of military health personnel with internationally agreed principles in military medical ethics (MME). In response, a number of authors have called for clarity on the principles and topics within the domain of MME. This complements an increased acknowledgment of the need for education in MME for military health personnel. Our paper utilizes bibliometric techniques to identify key themes in MME to inform the development of a curriculum for this subject.

**Materials and Methods:**

We designed a search strategy to find publications over the period January 1, 2000-December 31, 2020 in the domain of MME from the three databases, PubMed, Web of Science, and Scopus, using the search string (ethic* OR bioethics* OR moral*) AND military AND (medic* OR health*). We obtained a total of 1,115 publications after duplication removal. After exclusion based on topic, year, and study design, we analyzed a total of 633 publications using Scopus’s embedded analysis tool and the software VOSViewer. We generated a co-occurrence word map from the abstracts of each of the publications. We deduced themes of MME based on the clusters shown in the word map, and we categorized each publication into one of these themes to analyze the change of themes over time.

**Results:**

We observed a 10-fold increase in annual publications on MME between 2000 and 2020. The majority of papers were written by U.S. (72%) and UK (13%) authors, although a total of 15 countries were represented. After using VOSViewer to identify co-occurring keywords in titles and abstracts from these publications, nine themes were identified: biomedical research, care to detained populations, disaster/triage, mental health, patient-focused foundations, technology, dual loyalty, education/training, and frameworks. The relative proportion of each of these themes changed over the study period, with mental health being dominant by the end.

**Conclusions:**

This study has identified key themes that might inform the development of a curriculum for teaching MME. It is noticeable that the majority of themes cover MME from the perspective of professional practice on military operations; noting, the research and technology themes also pertain to the generation of knowledge for military operations. There were a limited number of publications covering practice in the non-deployed or garrison settings, and these were codified under the themes of “framework” and “dual loyalty”. The results are skewed toward English-speaking countries and exclude non-academic publications. Further work will search for other open-source information and non-English publications. To our knowledge, this exploratory bibliometric analysis on MME in the academic literature is the first of its kind. This article has demonstrated the use of bibliometric techniques to evaluate the evolution of knowledge in MME, including the identification of key themes. These will be used to support further work to develop a curriculum for the teaching of MME to military medical audiences.

## INTRODUCTION

The past two decades of conflict, especially in Iraq and Afghanistan, have led to public debate about the ethical professional practice by members of military health services.^1,2^ Appropriately, both Gross^3^ and Rochon^4^ have called for clarity from practitioners and scholars on the principles and topics within the domain of military medical ethics (MME) in 2013 and 2015, respectively. Later in 2015, the International Committee of the Red Cross published a consensus document in concert with the World Medical Association, the International Committee of Military Medicine, the International Council of Nurses, and the International Pharmaceutical Federation entitled “Ethical Principles of Health Care in times of Armed Conflict and other Emergencies”. The first principle of the document states “ethical principles of health care do not change in times of armed conflict and other emergencies and are the same as the ethical principles of health care in times of peace.” The document also asserts the principle that “the primary task of health care personnel is to preserve human physical and mental health and to alleviate suffering.”^5^

Although these principles are a step forward, they still need to be adapted into a military training context so that uniformed health care providers are educated in the ethical aspects of their role in order that they comply with International Humanitarian Law and the ethical standards set by their professional regulatin g bodies. Withnall and Brockie have proposed that education in military ethics should be an operational priority to all uniformed health professionals.^6^ This ethics education is vital to ensure that military healthcare providers act appropriately when discharging their dual professional obligations, both medical and military. A more thorough understanding of the moral challenges and ethical dilemmas encountered in military service will improve service members’ ability to identify and address ethical concerns in practice. Improving this ability became a priority for U.S. and UK Militaries following widespread attention and concern over violations of human rights of detainees in the early 2000s. However, as warfare has become more congested and unconventional in the subsequent 20 years, ethical dilemmas within MME have broadened, become more complex, and have received greater research interest. In response, in 2020, the U.S. DoD published an instruction outlining a code of medical ethics in its military health system that is now driving an educational program led by the Defense Medical Ethics Centre at the Uniformed Services University of the Health Sciences.^7^ Initiatives like this one should be underpinned by an evidence-based curriculum for teaching MME to military health professionals that establishes the baseline knowledge of MME and issues that might arise while in practice. To ensure the broad and relevant applicability of MME education, the profession of military medicine will benefit from a comprehensive overview of the ethical dilemmas arising in military health practice over the past 20 years. The purpose of this exploratory study, using bibliometric techniques, was to determine the MME topics and themes that have been covered in the academic literature in order to inform the development of a curriculum for teaching MME.

Bibliometric analysis is a technique that quantifies the bibliographic data (author, affiliation, journal, and citations) of a collection of publications and further draws relationships between these publications if they exist.^8^ A further application of bibliometric techniques to the text within publications is called text mining and is used to analyze unstructured data like text in abstracts and draw semantic relationships between the text to better classify themes within a body of literature.^9^ A bibliometric approach was chosen for this study because of the objectivity it provides compared to qualitative methodologies like phenomenology or grounded theory. Thomas et al. recommend a logical, objective component of the content creation within curriculum development, especially when there is the potential for disagreement between professionals and/or practitioners.^10^ Because debated opinions are the essence of ethical dilemmas and are often the topic of MME publications, this study utilizes mostly quantitative bibliometric approach to capture relevant debates over the past 20 years, which are essential content for military medical practitioners.

## METHODS

This study sought to identify all academic papers on the topic of MME published between January 1, 2000 and December 31, 2020 and then categorize them by date of publication, country of origin, and academic theme.

### Search Strategy

Three academic literature databases were searched: PubMed by US National Library of Medicine, Web of Science (WoS) by Clarivate Analytics, and Scopus by Elsevier. We used the search string, (ethic* OR bioethics* OR moral*) AND military AND (medic* OR health*) against titles, keywords, and abstracts. We found 2029 publications, with 530 from PubMed, 511 from WoS, and 989 from Scopus. Of note, we collected 53 publications that were non-English in origin but had translated titles and usually translated abstracts. This finding aligns with the fact that these databases predominantly collate English language publications.

### Screening Process

**FIGURE 1.**
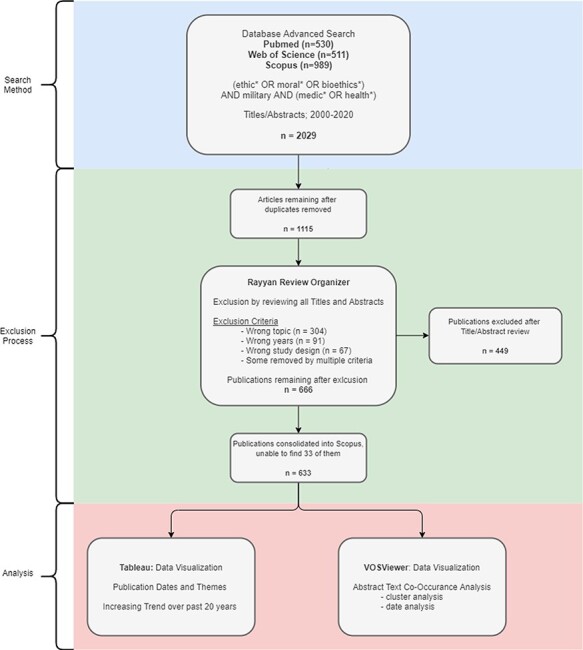
PRISMA flow diagram for the identification and selection of MME publications.^9^

After removing duplicates based on title and author pairings using the Excel add-in Ablebits (Version 2021.1.2588 by 4Bits Ltd.), we reviewed the remaining 1,115 publications by title and abstract against the following inclusion criteria using Rayyan literature review organizer (Qatar Computing Research Institute)^11^: (1) publication subject must be relevant to the years 2000-2020, (2) publication must be relevant to MME, specifically referring to a medical ethics topic concerning the military (as opposed to just providing the military as an example), (3) publication must focus on ethical implications of issues within military medicine (as opposed to referencing ethical approval for a clinical study about military medicine). M.B. informed the database selection and inclusion criteria, and Z.B. performed all collections of publications and inclusion evaluation. Following this exclusion process, a total of 666 publications met the criteria, of which 37 were non-English in origin but had abstracts and titles in English. Figure 1 depicts a flowchart of the search and screening process described above, in addition to the analysis of the remaining publications.

### Bibliometric Analysis

For the bibliometric analysis, we first consolidated all results into Scopus to aggregate data on affiliations and journals. We did this by manually searching Scopus for individual articles that were found by PubMed or WoS but not Scopus in the initial search. We could not find 33 of the publications on Scopus, so we conducted all analyses on just 633 publications rather than the full 666, losing primarily low relevance publications. The tools within Scopus allowed for data extraction of author information, affiliation information (both institution and country), publication dates, and publication journal.

We then utilized Tableau (Version 2020.4)^12^ to visualize these data, and we used the bibliometric software tool VOSViewer (Version 1.6.16 by the Centre for Science and Technology Studies at Leiden University)^13^ to visualize the text mining performed on the abstracts of the included publications. We uploaded the Scopus publication list into VOSViewer and performed a co-occurrence analysis on the text within the abstracts of all 633 publications, determining which words occurred most frequently across all papers. After text mining the abstracts, a total of 432 words were used at least 15 times throughout the text. To determine the most relevant words to include in the co-occurrence analysis, we first eliminated common verbs and nouns and then terms that are common to all academic publications and terms pertaining to demographics, occupations, or locations, reducing the number of words to 91. Finally, we conducted an internal consensus-building process, with the goal of narrowing the range of words to those whose presence could not be explained by anything except relevance to the topic of MME. This process used a survey that asked the authors of this article to include or exclude the remaining terms. Terms were then included if three or more individuals chose to include the term. We proceeded to the construction of themes based on a final list of 50 words. Z.B. performed data visualization in Tableau and VOSViewer. P.M. and M.M. were part of the process to select terms for inclusion in the VOSViewer map, and they both contributed significantly to the writing and revision of the introduction and discussion.

### Construction of Themes

**FIGURE 2.**
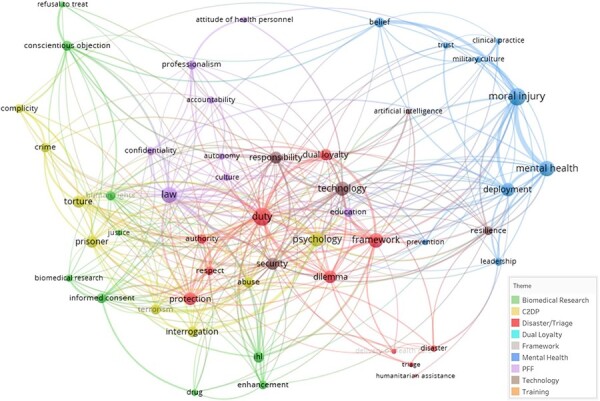
Cluster co-occurrence word map of the abstract text from all 633 publications included in the analysis. The clustering and mapping of each node was performed by VOSViewer using information about how often words were used in the same abstract as others and how often the words were used in conjunction with each other in a sentence or phrase.

The co-occurrence word map (Fig. 2, Results) provided clusters of words from the abstracts based on their relative relatedness, as quantified by VOSViewer.^14^ The relatedness algorithm takes into account how many times words are used within the same abstract, in addition to how closely these words are used together within that abstract. The higher the relatedness between two terms, the smaller the distance will be between those two terms on the co-occurrence map. Therefore, the repetition of the above process for every word leads to spatial cluster formation as seen in Figure 2. We derived general topics of MME in academic publications from the common terms that arose in each cluster of the co-occurrence map generated by VOSViewer. These themes were then combined with the publication dates of papers that focused on a specific theme. This helped to generate a graph of thematic changes in MME from the year 2000 to 2020.

## RESULTS

### Overview of Publication Data

The most prominent trend that arose in the literature review was the increase in publications on MME from 4 or 5 per year to 55+ from 2017 onwards. Although there is a long-term upward trend of publications, significant spikes have occurred from 2005 to 2008 (+220%) and from 2012 to 2018 (+280%), with a small dip from 2009 to 2011, where the number of publications fluctuated up and down.

From the 633 collected publications, we extracted aggregate information on authors, affiliations, and journal titles. These publications represented a total of 159 authors from 15 different countries. A rank order of the most prevalent authors, countries, affiliations, and journals is shown in Table I.

**TABLE I.** Top 15 Rank Order of Authors, Countries, Affiliations, and Journals in the Reviewed Publications

Rank	Author	Country	Affiliations	Journals
1	J.M. Currier (11)	USA (453)	U.S. VA Medical Centers^a^ (81)	*Military Medicine* (60)
2	H.G. Koenig (11)	United Kingdom (78)	U.S. Active Duty Military Health Centers^b^ (53)	*Journal of Royal Army Medical Corps* (38)
3	N. Greenberg (10)	Canada (40)	King’s College London (18)	*Journal of Medical Ethics* (12)
4	M.L. Gross (9)	Australia (24)	Duke University (12)	*Military Psychology* (11)
5	E.J. Teng (8)	Israel (20)	Queen Elizabeth Hospital, Birmingham (12)	*American Journal of Bioethics* (9)
6	D. Ames (6)	Netherlands (17)	University of South Alabama (12)	*Aviation Space and Environmental Medicine* (7)
7	B.T. Litz (6)	China (8)	Baylor University (10)	*Journal of Law Medicine and Ethics* (7)
8	M. Pearce (6)	Iran (7)	King Abdulaziz University (10)	*Journal of Military Medicine* (7)
9	N.A. Youssef (6)	Germany (5)	Harvard University (8)	*Journal of Military Ethics* (6)
10	G.J. Annas (5)	South Africa (4)	Boston University (8)	*New England Journal of Medicine* (6)
11	W.B. Johnson (5)	Turkey (4)	UCLA (8)	*Psychological Trauma* (6)
12	M.S. Kopacz (5)	Brazil (3)	Ningxia Medical College (8)	*Traumatology* (6)
13	N.T. Fear (4)	Switzerland (3)	University of Haifa (8)	*Cambridge Quarterly of Healthcare Ethics* (5)
14	C.R. Figley (4)	France (2)	University of Oxford (7)	*Medicine, Conflict and Survival* (5)
15	T.M. Gibson (4)	Italy (2)	U.S. Medical College of Georgia (7)	*BMJ Open* (5)

a The U.S. VA Medical Centers are comprised of multiple centers including Michael DeBakey VA Medical Center, VA Palo Alto Health Care System, Greater Los Angeles VA Medical Center, Durham VA Medical Center, VA Boston Health Center, and the VA Midwest Health Care Network.

b U.S. Active Duty Military Health Centers are comprised of the Air Force School of Aerospace Medicine, Air Force Institute of Technology, Wilford Hall Medical Center, Brooke Army Medical Center, Walter Reed National Military Medical Center, and the U.S. Naval Academy.

We observed that majority of affiliations were civilian as opposed to military. 67% of the affiliations were civilian universities, hospitals, or other institutions. Furthermore, of the 209 military-affiliated publications, nearly 85% were from the USA.

### Thematic Analysis

This section combines the results of the thematic analysis with the rise in publications within MME to demonstrate the change in themes from 2000 to 2020. We used VOSViewer to generate the cluster co-occurrence word map, shown in Figure 2. This map shows the 50 most used terms in reference to MME in the titles and abstracts of the 633 publications included in this review. As described in the Methods sections and other publications,^14^ VOSViewer determines the spatial distance (mapping) and color-coding (clustering) of each node using a unified approach of related formulas.

From this cluster analysis, we created a hierarchy of the words to elucidate themes from the map itself. We used the identified clusters as reference guides for forming themes, although the chosen themes overlap the colored clusters in some areas. By starting with the clearly differentiated edges of the word map, we worked toward the center of the map to create the following themes: biomedical research (green), care to detained populations (C2DP) (yellow), disaster/triage (red), mental health (blue), patient-focused foundations (PFF) (purple), technology (brown), dual loyalty, education/training, and frameworks. The final three themes listed are not listed by color because they are not differentiable by their color in the word map. Instead, those three themes arose from the remaining publications after we had classified each publication into one of the six themes that are clearly identified by color in Figure 2. These last three themes are also corroborated by the fact that they are centrally located in word map. They clearly have distinct definitions and meanings but are often multifaceted topics, so they are more closely related to words in all areas of the co-occurrence map.

**FIGURE 3.**
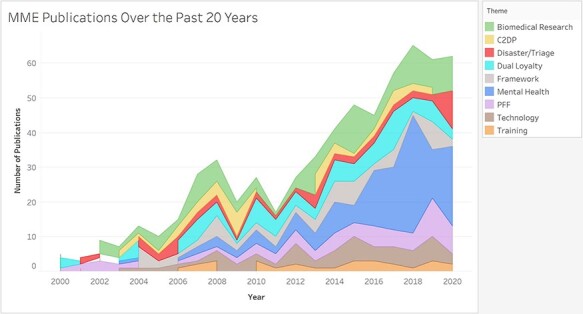
Discrete area chart exhibiting the demonstrable increase in publications from 2000 to 2020. The previously derived themes are included in the legend and are differentiated in the area chart itself. Reference dates are overlaid; these events are hypothesized to have influenced the increase in overall number and topical number of publications.

To complement and build upon this thematic identification, we classified each publication into one of the nine themes drawn from Figure 2. Biomedical research includes publications that addressed ethics of performance enhancements through drugs or training regimes, trial recruitment procedures in the military, and informed consent specifically for research. Care to detained populations includes publications that addressed medical attention to captive detainees. Disaster/triage includes publications that addressed medical care in natural or man-made disasters, including rapid humanitarian responses and triage prioritization. Dual loyalty includes publications that addressed dilemmas in duties and obligations of those who are considered both military and healthcare professionals. The framework includes publications that addressed many aspects of MME or put forward a framework for implementing MME. Mental health includes publications that addressed moral injury, PTSD, or other mental health illness or intervention. Patient-focused foundations include publications that addressed basic principles in MME such as autonomy, confidentiality, and beneficence but in a military context. Technology includes publications that addressed the use of robotics, drones, or artificial intelligence in military medicine. Finally, training includes publications that addressed the medical aspects of ethics education used within the military context. The classification of publications in these nine themes per year is shown in Figure 3. This area chart shows the relative increase in individual themes over the past 20 years, overlaid onto the overall increase in publications on MME shown in Figure 1.

The theme identification and subsequent publication classification revealed a sharp increase in publications concerning Mental Health starting in 2014, alongside smaller, but steady, increases in technology, biomedical research, and PFF MME publications over the past 20 years. There has been a consistent number of training, dual loyalty, and framework publications since 2006. Finally, there have been two periods of time for increases in C2DP publications, 2005-2009 and 2013-2018.

## DISCUSSION

This discussion section includes an examination of the suitability and role of methodology for curriculum development, followed by an analysis of the findings in Table I, and an interpretation of the thematic mappings (Fig. 2) and theme growth over time (Fig. 3), and then, a consideration of the limitations and implications on the findings, and finally the current outlook on future work.

According to Thomas et al., the first stage of curriculum development in medical education is problem identification and assessment of the learners’ needs against measures of the current state of knowledge in the field.^10^ A key component of the needs assessment is obtaining information on needs, which can be done primarily in three ways: a review of available knowledge, use of experts in multi-stakeholder consultation and consensus-building, and the collection of new information.^10,15^ In the review of available knowledge, it is necessary to codify the current state of knowledge through an analysis of the published literature. Bibliometrics has become an increasingly important tool that can summarize large volumes of academic literature to provide an overview of the state of knowledge in a particular field.^16^ This article presents the first bibliometric analysis on the subject of MMEs in academic publications, which functions as a tool for problem identification and a review of available knowledge. By characterizing MME into a collection of themes, this study defines the themes that should be contained within a curriculum for teaching MME, which is a key question in problem identification. This defined scope, along with the typified literature, will provide an underlying structure and the content with which an expert panel can build consensus around MME dilemmas.

When looking at the pool of available knowledge in the academic literature, Figure 3 visually shows that the overall MME publication rate has increased substantially over the past two decades, highlighting the rising importance of the topic and corroborating the need for a systematically developed curriculum. This positive trend probably reflects the increased collective experience of providing medical support to combat operations by military health personnel from the countries of the authors of the publications. However, the increased military medical experience cannot be the only explanation because of the preponderance of authors from civilian institutions. This imbalance is partially explained by a significantly larger number of civilian academic researchers, but it also demonstrates civilian concern over and influence on ethical practice by military personnel,^17^ especially as military personnel may be constrained by military law from publicly criticizing the armed forces.^18^ Looking at the country affiliation of the MME publications, the majority have originated from the USA and the UK. This prominence could reflect the relative scale of military commitments to combat operations for these two countries, but because only English language databases and English search terms were used, the significant bias against countries with native languages other than English is the explanatory factor most responsible.

This study uses VOSViewer as a tool to identify and illustrate key terminology and their relationship across the literature as shown in Figure 2. These were combined into themes through a survey across the research team to build an internal consensus of pertinent MME terms. These themes were mapped against time in Figure 3. This is a useful method to determine themes and topics for inclusion within a curriculum for MME, particularly because the objectivity of the tool reduces the likelihood of bias attributable to the researchers. However, it is important to note that it is biased by the collective perspective of publication authors because of the quantitative methodology it uses. This bias needs to be balanced by reference to a pool of subject matter experts to further develop an MME curriculum.

This thematic categorization also collates publications ready for a more detailed literature review of each theme. The themes “dual loyalty” and “PFF” are the most enduring and should provide the underlying principles in an MME curriculum. The dual loyalty publications constitute the heart of the competing obligations of two professions^19–22^ that often have opposing priorities of national and personal security. The disaster/triage theme is a prime example of opposing priorities for access to clinical care imposed by dual loyalties because warfare complicates the objectivity of standard casualty care by introducing enemies, allies, prisoners, non-combatants, non-state actors, etc.^23,24^ Many of the PFF publications focused on the limits of confidentiality in a hierarchical military setting^25–29^ and justice in medical treatment with respect to gender and sex.^30–33^ Although there was some discussion of patient autonomy and informed consent regarding soldiers and vaccinations^34^ within the PFF publications, the majority of publications concerning informed consent fell under the biomedical research theme.

The biomedical research theme covers both the topics for biomedical research in military research and development programs, like neurological^35–38^ and physical^39–41^ enhancement and the compliance of this research with military subjects with ethical practice and regulations.^42–45^ Although not all military health professionals will conduct biomedical research, the basic principles should be taught to all healthcare providers to increase internal accountability among those conducting military biomedical research. We separated this theme from the theme of “technology” in order to differentiate between biomedical research and the ethical implications of the use of existing or new technologies for military purposes, such as novel uses of electromagnetic radiation^46^ or biological agents^47,48^ as bioweapons or drone technology used for medical surveillance or telehealth.^49–51^

Despite Singh’s 2003 advice^52^ to the U.S. Government to ensure necessary protections to detainees, the mistreatment of detainees following Iraq and Afghanistan led to the rapid increase in “C2DP” publications from 2005 to 2009, specifically with respect to prisoners held at Abu Ghraib and Guantanamo Bay. In 2014, a further increase of publications followed U.S. Congressional Research Reports on the same issue of detainee treatment.^53^ The combination of this surge of publications in this theme and the parallel increase in publications from civilian institutions further demonstrates the desire for civilian oversight of the military. The heaviest criticism and subsequent publications have explored physician responsibility to report mistreatment^54,55^ and handling of life-sustaining treatments, such as “force-feeding” those under hunger strikes.^56,57^

The mental health theme also increased following Iraq and Afghanistan as a new generation of soldiers battled PTSD^58,59^; however, the rapid increase in 2012 probably reflects an increase in the use of the term “moral injury” as an explanation for the mental health consequences of undertaking or witnessing acts that conflict with an individual’s ethical or moral beliefs.^60–62^ Aligning with Farnsworth and Capone, we anticipate that preparatory training and education in the types of scenarios that may lead to moral injury could provide some preventative protection.^63,64^ In regard to the Training theme, a consistently low number of publications are indicative of a lack of attention to proper curriculum development for MME education and training. Although the U.S. DoD has formalized a Defense Medical Ethics Center and developed a code of MME,^7^ there has been less done to translate the code into an educational MME curriculum or appropriate platform, further highlighting the value of this present study and future work.

In addition to developing these themes, we also noticed that there seem to be gaps in the literature. There were a limited number of publications covering practice in the non-deployed or garrison settings or specific MME dilemmas from the perspectives of nursing or allied health professions. The deployed military setting has garnered significant public attention and subsequent criticism, alongside increased publication rates. This realization might suggest that military medical professionals would benefit from greater self-interest in MME research areas that do not draw as much public attention, like a non-deployed setting or role-specific dilemmas.

This study has successfully identified key themes within the MME academic literature, and it has also demonstrated a repeatable methodology with which to derive themes from a large body of literature. Given the limitations outlined below, we will revisit this methodology with various other sources to get a more complete picture of MME. The three significant limitations were the lack of other languages besides English, the lack of gray literature, and finally, the lack of analysis into individual themes.

We conducted this bibliometric analysis on academic publications in the three databases of PubMed, WoS, and Scopus with titles and abstracts in English. Our choice of databases excluded non-academic publications, particularly policy documents, although these might have been the subject of analysis of the academic papers identified. Furthermore, these databases have been shown to miss non-English origin publications in comparison to a broader search engine like Google Scholar.^65,66^ We plan to employ a similar method to identify relevant open-source publications using an internet search engine and to search non-English language databases using researchers with appropriate language skills. After collating these additional papers, the process of thematic categorization will be repeated to determine if further themes or perspectives can be identified from authors from other countries or writing in other languages. This process will form the basis of further work for curriculum development of educational packages in MME. In addition to broader resources, the subsequent curriculum development would benefit from a deeper exploration of each theme that we derived from the VOSViewer map. Each of the 633 publications has already been sorted into respective themes to create Figure 3, so the application of a qualitative approach like phenomenology with a coding tool like NVivo to the collections of papers within each theme would be useful for further defining MME dilemmas in relation to curriculum development and for targeted case studies or policy investigation in particularly themes.

## CONCLUSION

The topic of MME has become more prominent in the academic literature over the first 20 years of the 21st century. This has been reflected by the call for specific education in this field by a number of authors. This article has demonstrated the use of bibliometric techniques to evaluate the evolution of knowledge in MME, including the identification of nine key themes: biomedical research, C2DP, disaster/triage, mental health, PFF, technology, dual loyalty, education/training, and frameworks. This analysis is biased toward English language academic publications, reducing the influence of non-English language authors and gray literature. The themes are weighted toward deployed healthcare/operational issues with a relative under-representation of garrison healthcare and non-physician professions. It is intended to extend the methodology to identify and collate additional literature resources, including gray literature and other languages. It is also intended to deepen the analysis by conducting literature reviews of publications within each theme. This information will be utilized to inform a board of subject matter experts to build consensus on ethical issues in each of these themes. This work will form the foundations of a curriculum for MME, pending the evaluation of education techniques for the implementation of the curriculum.
